# Familial Dilated Cardiomyopathy Caused by a Novel Frameshift in the *BAG3* Gene

**DOI:** 10.1371/journal.pone.0158730

**Published:** 2016-07-08

**Authors:** Rocio Toro, Alexandra Pérez-Serra, Oscar Campuzano, Javier Moncayo-Arlandi, Catarina Allegue, Anna Iglesias, Alipio Mangas, Ramon Brugada

**Affiliations:** 1 Medicine Department, School of Medicine, Cadiz, Spain; 2 Cardiovascular Genetics Center, IDIBGI, University of Girona, Girona, Spain; 3 Department of Medical Sciences, School of Medicine, University of Girona, Girona, Spain; 4 Cardiac Genetics Unit, Hospital Josep Trueta, University of Girona, Girona, Spain; University of Tampere, FINLAND

## Abstract

**Background:**

Dilated cardiomyopathy, a major cause of chronic heart failure and cardiac transplantation, is characterized by left ventricular or biventricular heart dilatation. In nearly 50% of cases the pathology is inherited, and more than 60 genes have been reported as disease-causing. However, in 30% of familial cases the mutation remains unidentified even after comprehensive genetic analysis. This study clinically and genetically assessed a large Spanish family affected by dilated cardiomyopathy to search for novel variations.

**Methods and Results:**

Our study included a total of 100 family members. Clinical assessment was performed in alive, and genetic analysis was also performed in alive and 1 deceased relative. Genetic screening included resequencing of 55 genes associated with sudden cardiac death, and Sanger sequencing of main disease-associated genes. Genetic analysis identified a frame-shift variation in *BAG3* (p.H243Tfr*64) in 32 patients. Genotype-phenotype correlation identified substantial heterogeneity in disease expression. Of 32 genetic carriers (one deceased), 21 relatives were clinically affected, and 10 were asymptomatic. Seventeen of the symptomatic genetic carriers exhibited proto-diastolic septal knock by echocardiographic assessment.

**Conclusions:**

We report p.H243Tfr*64_*BAG3* as a novel pathogenic variation responsible for familial dilated cardiomyopathy. This variation correlates with a more severe phenotype of the disease, mainly in younger individuals. Genetic analysis in families, even asymptomatic individuals, enables early identification of individuals at risk and allows implementation of preventive measures.

## Introduction

Dilated cardiomyopathy (DCM) is a heart disease with a prevalence of 1:2500 in the general population. DCM is characterized by left ventricular (LV) enlargement and subsequent systolic dysfunction, but dilatation of both ventricles may occur. This disease presents a wide range of symptoms; some cases are asymptomatic, while others exhibit severe heart failure (HF) leading to cardiac transplantation [1]. Additionally, onset of symptoms may occur at any age [2]. Familial DCM accounts for nearly 50% of all cases [3], and more than 250 pathogenic variations have been associated with the disease. These variations are located in nearly 60 different genes, which mainly encode for sarcomeric, cytoskeletal, and desmosomal proteins [1]. Almost 70% of familial cases have been attributed to a known genetic variation [3, 4]. Variants of the *TTN* gene are most prevalent, identified as causal in nearly 40% of cases [3]. Variations in the *LMNA* gene (10%) as well as *ACTC1*, *BAG3*, *RBM20*, *MYBPC3*, *MYH6*, *MYH7*, *TNNT2*, *TPM1*, and *SCN5A* (2–5%) account for most of the other known causal genetic alterations [3, 5]. Most of the known variations are single nucleotide variants. In addition, copy number variation (CNV) has recently been associated with DCM, but with a low incidence [6]. Several of the marginal disease-related genes have also been associated with diseases that cause sudden cardiac death (SCD), such as hypertrophic cardiomyopathy, arrhythmogenic cardiomyopathy, and left ventricular noncompaction [2].

Recent reports have linked DCM to variations in *BCL2-associated athanogene 3* (*BAG3*) [7, 8], which belongs to a family of co-chaperones whose BAG domains bind the ATPase domain of heat shock protein 70 to inhibit apoptosis [9]. Hence, DCM-associated *BAG3* variations may promote apoptosis of myocytes [10, 11]. A recent study indicates that individuals carrying *BAG3* variations that encode a truncated product exhibit delayed onset of symptoms [12]. Despite evidence of many causal variations, the pathophysiologic mechanisms associated with severe DCM phenotypes remain to be clarified. Here, we performed a comprehensive phenotype-genotype correlation analysis in a large Spanish family with many individuals exhibiting DCM. Our genetic screening identified a novel variation responsible for the familial disease.

## Methods

Written informed consent was obtained from all individuals studied. The study was approved by the ethics committee of the Hospital Josep Trueta (Girona, Spain), following the Helsinki II declaration. Detailed clinical data were obtained from each subject, including family history, age of presentation, initial symptoms of HF, physical examination, serum creatine kinase, 12-lead electrocardiogram (ECG), transthoracic echocardiography (TTE) using tissue Doppler imaging (TDI), ECG-Holter monitoring, when appropriate, cardiac-magnetic resonance imaging (CMRI), as already published [13].

Genomic DNA was extracted from whole blood (PerkinElmer Inc). DNA was extracted from paraffin-embedded heart tissue by using BiOstic FFPE Tissue DNA Isolation Kit (MoBio Laboratories Inc). DNA was fragmented and library preparation was performed according to the manufacturer’s instructions (SureSelect XT Custom 0.5–2.9Mb library, Agilent Technologies Inc). Next-generation sequencing (NGS) was performed on the MiSeq System (Illumina). We analyzed the 55 most prevalent genes involved in SCD-related pathologies [14] (*ACTC1*, *ACTN2*, *ANK2*, *CACNA1C*, *CACNB2*, *CASQ2*, *CAV3*, *CRYAB*, *CSRP3*, *DES*, *DMD*, *DSC2*, *DSG2*, *DSP*, *EMD*, *FBN1*, *GLA*, *GPD1L*, *HCN4*, *JPH2*, *JUP*, *KCNE1*, *KCNE2*, *KCNH2*, *KCNJ2*, *KCNQ1*, *LAMP2*, *LDB3*, *LMNA*, *MYBPC3*, *MYH6*, *MYH7*, *MYL2*, *MYL3*, *MYOZ2*, *PDLIM3*, *PKP2*, *PLN*, *PRKAG2*, *RYR2*, *SCN4B*, *SCN5A*, *SGCA*, *SGCB*, *SGCD*, *TAZ*, *TCAP*, *TGFB3*, *TGFBR2*, *TNNC1*, *TNNI3*, *TNNT2*, *TPM1*, *TTN*, and *VCL*). The results were analyzed with GEM III [15] and SAMtools v.1.2 [16] software. Post-mortem DNA was analyzed by Sanger sequencing (see below) due to low quality/integrity.

Genetic variants and allelic frequencies were scored based on dbSNP, Exome Variant Server, 1000 Genomes, Ensembl, Human gene mutation database (HGMD® Professional) from BIOBASE Corporation, Exome Aggregation Consortium (ExAC), and an in-house database. Variants were annotated and allelic frequency was consulted using the same databases. *In silico* pathogenicity of novel genetic variations were consulted in CONDEL, PROVEAN, and Mutation Tester. Alignment among species was also performed using the Universal Protein Resource database.

Non-common (Minor Allele Frequency–MAF- < 1%) genetic variants were confirmed by Sanger method. Molecular screening of the novel variation was performed in 276 Spanish control subjects (552 alleles) not related to any patient. Familial cosegregation of rare genetic variants was also performed using Sanger technology.

Sanger sequencing of all coding exons and flanking intronic sequences of *BAG3* (NM_004281.3), *RBM20* (NM_ NM_001134363), and *TMPO* (NM_ NM_001032283) were performed. It was amplified by PCR, purified, and directly sequenced in both directions. SeqScape Software v2.5 (Life Technologies) was used to compare results with the reference sequence from Hg19.

Paraffin myocardium sections of 10 μm thick were obtained with a Leica RM2235 (Leica Microsystems GmbH) and mounted on Super Frost slides (Thermo Fisher Scientific Inc). Random representative slices were stained with hematoxylin-eosin, picro-Sirius Red, and Masson’s Trichrome (standard protocol, Sigma-Aldrich Química,S.L.). After washing, slides were dehydrated in alcohol, cleared in xylene, and covered with DPX (44581, Fluka). A minimum of three labeled slices were studied through a Nikon Eclipse 50i microscope interfaced to a DS-2Mv camera and a HP4600 PC. Pictures of representative areas were taken using the NIS-Elements BR3.0 software (Nikon).

In mutant carrier as well as in control samples, total protein from embedded tissue was extracted according to the manufacturer's instructions (Qiagen). To perform an electrophoresis in a 10% acrylamide SDS-PAGE gel, 40 μg of total protein were used. Proteins were transferred onto PVDF membrane and blocked for 1 hour in 5% non-fat milk in 1% tween20® phosphate-buffered saline pH 7.4 (PBS-T). The membrane was incubated with anti-BAG3 (DAB0330, Abnova) or anti-α-actin (A2066, Sigma-Aldrich Química, S.L.) antibody at 1:2000 dilutions overnight at 4°C. After washes in PBS-T the membrane was incubated with anti-rabbit-HRP secondary antibody for 1 hour (1:10000) at room temperature. Then, membrane was incubated with Pierce ECL (Thermo Fisher Scientific Inc) and revealed with X-film. The signal for BAG3 and α-ACTIN was measured by densitometry using Image software (NIH).

## Results

**Fig 1:**
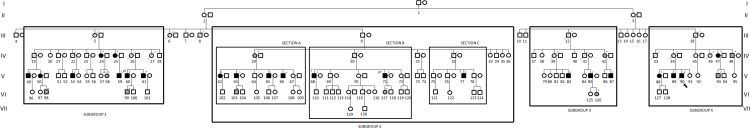
Generations I to VII are indicated in the image sides. Each individual of direct family linage is identified with a number. The index case is indicated by arrow (V-90); mutation carriers are represented with a **+**. Symptomatic patients are shown in black, asymptomatic patients are shown in gray, slashes indicate individuals who are deceased. T: transplanted; p-T: Pre-transplant stage.

**Fig 2:**
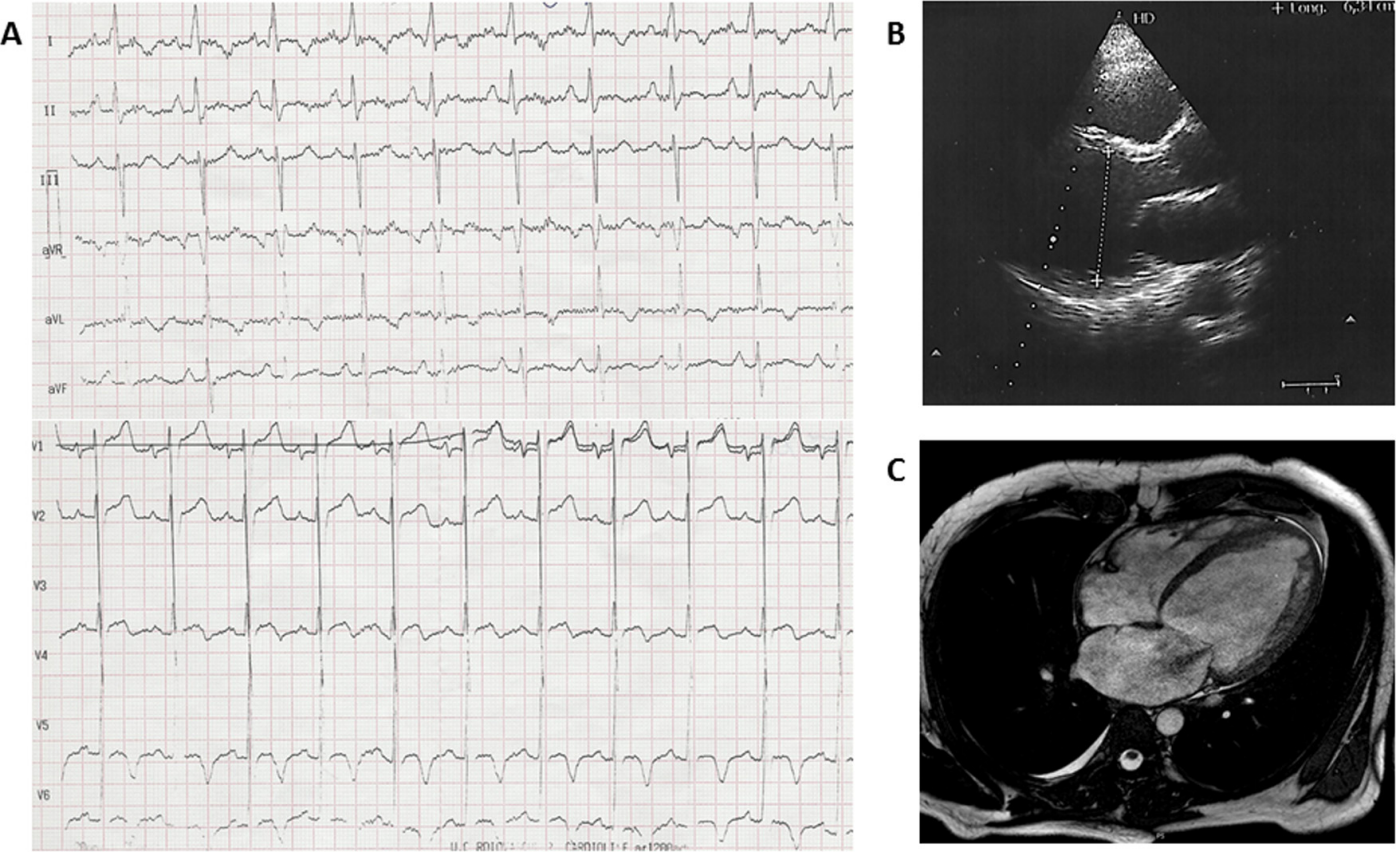
Representative clinical test in diagnosed patients. A. Electrocardiogram of adult with DCM. B. Transthoracic echocardiogram of patient with DCM. C. Cardiac MRI showing ventricle alterations diagnostic of DCM.

**Fig 3:**
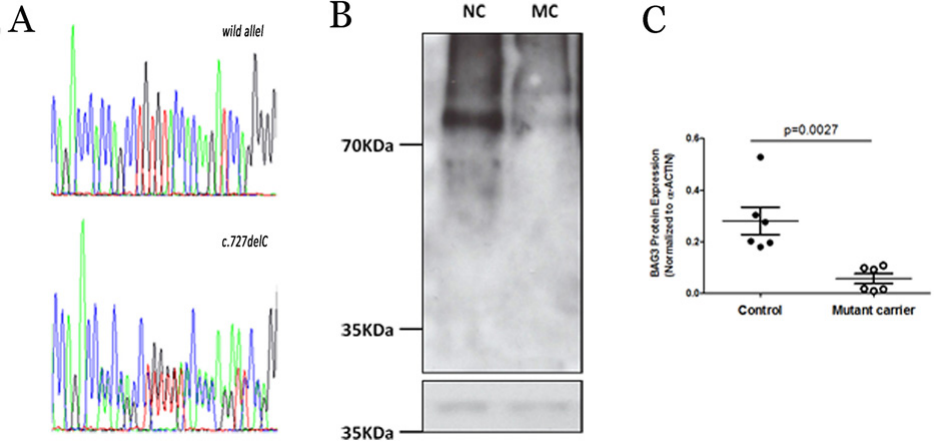
A. Chromatograms illustrating novel *BAG3* genetic variant found, p.H243Tfr*64_*BAG3*. B. Representative western blot showing BAG3 and the load control α-ACTIN. C. Quantification of BAG3 protein levels in control and mutant carrier. Values are shown as BAG3 signal normalized to α-ACTIN levels to each replicate, and horizontal lines represent mean±SEM (standard error) for each sample. Statistical analysis was performed using two-tailed Mann-Whitney test.

Our study included a total of 130 members of a seven-generation Spanish family diagnosed with DCM (Figs 1 and 2). One hundred living members were clinically and genetically assessed, although our index case was a deceased man (V-90). Subjects ranged in age between 5 and 73 years. Genetic analysis of the index case (V-90) using NGS did not identify any variation that was either novel or previously reported as pathogenic/potentially pathogenic. Subsequently, a traditional genetic analysis of *BAG3*, the most prevalent gene associated with DCM not included in our NGS panel, was performed using Sanger method. A novel variation, a cytosine deletion at base position 727 (c.727delC), was identified (Fig 3). It induces a frame shift in amino acid 243 (Histidine) with a subsequent premature STOP codon (p.H243Tfr*64). This mutation has been not identified in the ExAC database. *In silico* prediction (Mutation Tester) reported a deleterious effect. We tested a novel variant in all alive members of the family, being 31 (23.8%) of them positive. From 32 genetic carriers (31 alive and 1 post-mortem), twenty-one (65.6%) were males. Other twenty-one (65.6%) showed clinical symptoms associated with the disease and the mean age at clinical diagnosis was 43.1 years (Table 1) (Fig 2).

**Table 1:** Echocardiographic findings in patients carrying the genetic variant associated with DCM. Gender: M, male; F, female. Symptoms: P, palpitations; T, transplanted; p-T, Pre-transplant stage; p-S, Presyncope; S, Syncope; SHF, sudden heart failure; (-) means no dyspnea. New York Heart Association Functional Classification is indicated by a rank between 1 and 4. LVEF indicates left ventricle ejection fraction; TDLVD, telediastolic left ventricle diameter; LAA1, left atrial volume; E/A ratio, relation between E-wave and A-wave in Doppler. Knock in myocardium is indicated by presence (1) or absence (0).

Sample	Sex	Age	Symptoms	LVEF	TDLVD	LAA1	E/A ratio	knock
V-88	F	39	-	52,0	53,0	17,0	0,89	1
V-89	M	38	DII	59,2	50,0	20,9	0,87	1
V-87	M	32	P	51.2	58.2	16,7	0.72	1
IV-47	F	54	DII	59,3	43,0	16,0	0,62	0
V-85	M	45	DII	64,0	54,4	23,3	0.95	0
V-83	M	45	DII	39,1	64,6	21,2	1,10	1
V-62	F	50	DII-III	38,6	57,8	17,9	0,88	1
V-63	M	48	DIII	38,4	60.1	21,2	0,90	1
V-65	M	45	P	39,2	62.3	19,4	0,71	1
V-66	M	37	P	45,0	59,0	21.0	1.24	1
V-68	M		T					
V-71	M	49	DIII, p-T	18,0	73,0	31,8	2,47	1
V-77	M		T					
V-49	M	44	p-S	48,0	63,0	21,7	1,29	1
V-50	F	43	P	57,3	59,3	19,3	1,15	1
V-53	M	29	DII	45,2	61,3	17,2	1,12	1
IV-24	F	65	DII,S,P	38,8	59.8	22,0	0,88	1
IV-25	F	72	DII-III,P	35,0	62.2	12,0	1,66	1
V-59	M	42	DII	42,0	62,0	17,5	0,79	1
V-60	M	36	SHF, p-T	27,0	69,8	17,2	1,37	1
V-61	M	35	P, DI	25,4	58,7	16,1	1,74	1

For ease of clinical reporting, we divided the family into subfamilies (Fig 1). Notably, all symptomatic relatives carried the mutation, and none of the symptomatic genetic carriers was younger than 20 years old.

**Fig 4:**
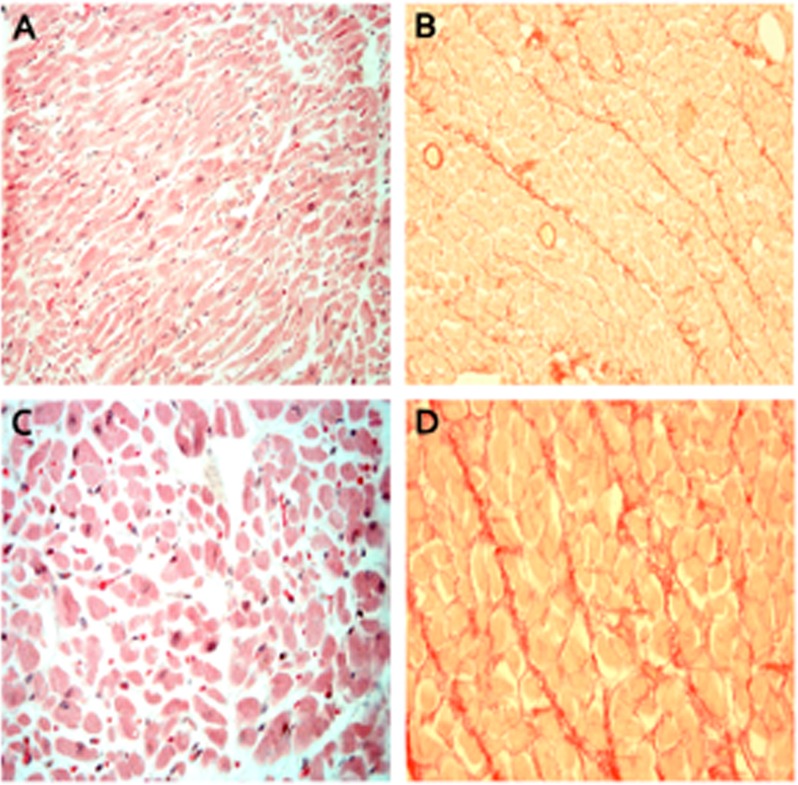
Microscopic images of post-mortem tissue. Pictures showed myofibrillar loss (A and C) and mild interstitial fibrosis (B and D). Nuclear pleomorphism and cellular atrophy is also observed (C). Hematoxylin-eosin labeling (A and C), and picro-Sirius Red labeling (B and D). Scale: A and B (200x) C and D (400x).

The proband (V-90) belongs to subgroup 4. He passed away at age 31 from acute cardiac failure. He presented with a respiratory infection with dyspnea, fever, and expectoration. Cardiac enzymes were within normal levels (Troponin I: 0.1 ng/mL and Creatinin Kinase 79 U/L). He also showed leucocytosis with elevated C-reactive protein (1.60 mg/dL). One month later, he came back with a new episode of dyspnea. An echocardiogram was performed showing a dilated LV (62 mm) with severely depressed systolic function (EF < 20%). He had a restrictive pattern (E/E´:25) and moderate pulmonary hypertension with slightly dilated RV with lower-limit function (TAPSE 16 mm). A pulmonary embolism was ruled out. He passed away ten days later. Autopsy data confirmed a dilated LV. The total weight of the heart was 713 g, with a global transversal diameter of 16 cm, anteroposterior diameter of 12 cm, and longitudinal diameter of 14 cm. An augment in total heart weight was observed, mainly due to enlargement of 9 cm in LV cavity. Left ventricular wall thickening was within normal range. No thrombus was detected in the atria. Microscopic analysis identified polyploidy and diffuse interstitial fibrosis but without signs of myocarditis or ischemic disease (Fig 4).

DNA extracted from post-mortem tissue identified the frameshift variation in the *BAG3* gene. To confirm the expected BAG3 protein reduction in the mutant carrier, we performed a western blot analysis. We observed a qualitative and quantitative reduction of native BAG3 in the mutant carrier tissue in comparison to a control sample (Fig 4B and 4C).

Seventeen family members died prematurely, three of them of non-cardiac cause (III-6, III-8, IV-41) and fourteen of them including the proband because of a sudden heart failure, (V-90, IV-44, IV-43, IV-46, III-18, III-12, IV-39, IV-30, IV-32, III-5, IV-19, IV- 22, IV-26, IV-27).They had an average age of 44.76 years-old, and 8 (58.8%) of them were males. Genetic analysis was not performed in any of these cases except the proband due to the lack of sample.

The asymptomatic mutation carriers in this family were 10 (5 were males) with a middle age of 35 years-old (IV-29, IV-42, IV-48, V-58, V-93, VI-98, VI-99, VI-103, VI-117, and VI-126). In all of them were performed a clinical, ECG and echocardiographic evaluation that was negative.

From twenty-one symptomatic mutation carriers (shown in Table 1), only two patients (IV-47, V-85) complained of functional class II dyspnea without any other echocardiographic impairment. Besides, the proband sister, V-88, who did not referred any cardiac symptom, showed diastolic pattern type I according to TDI despite her young age. The proband brother (V-89) complained of functional class II dyspnea and had a similar echocardiographic pattern. According to cardiovascular imaging thirteen (61.9%) patients, ten (76.9%) males with a mean age of 45.9 years old, showed dilated LV and depressed LEVF lesser than 48%. Interestingly, 17 of 19 echocardiograms performed in the symptomatic group showed a proto-diastolic septo-apical knock, non-related with conduction impairment neither other cause that justifies its presence. None of the positive asymptomatic carriers showed this septal knock. Two (9.5%) of the alive patients have been transplanted (V-68, V-77) and two (9.5%) are in a pre-transplant stage (V-71, V-60).

## Discussion

We report a large family diagnosed with DCM and exhibiting significant history of HF and cardiac transplant. Genetic analysis identified a novel frameshift (p.H243Tfr*64) genetic variation in *BAG3* that is segregating in all affected family members. Therefore, the novelty of the variant, the production of a truncated protein, deleterious *in silico* prediction, and positive family segregation suggest the pathogenicity of this novel variant in *BAG3* in this family with DCM.

Our results agree with reported cases of familial DCM with HF due to alterations in the BAG3 protein [12, 17]. Furthermore, we observed a significantly reduced level of BAG3 in the heart of a mutation carrier who died as a consequence of heart failure, in the same way as Feldman et al. [17]. Recent reports assert that reduced protein level is not related to mRNA levels [18], but BAG3 levels and BAG3 antibodies show an increase in serum of patients with HF [19, 20]. Together, these findings suggest that further studies are needed to define the cellular mechanisms that regulate protein levels of BAG3 in patients with cardiac HF.

The mean age onset of the DCM phenotype in this family was in the fourth decade; this is young according to widely published information about this disease [7], although the appearance of DCM with severe dysfunction is more remarkable in young patients. Nevertheless, it is difficult to establish its chronology, such as in LMNA cases [14].

The disease severity varied considerably: 2 members of this family had heart transplant and 2 are in a pre-transplant stage, 14 died with advanced HF and some of them died while waiting for cardiac transplantation. Ten mutation carriers were clinically and echocardiographic asymptomatic. This group included all children who were asymptomatic, and also some adults. The clinical findings observed in this family correspond to an incomplete penetrance of genetic variant expression. This phenotypic variability has been described previously [17].

TTE has proven useful for diagnosis, although young asymptomatic carriers, which included all children, do not show any alteration on TTE or tissue Doppler. In addition, ECG was normal in asymptomatic genetic carriers. TDI is useful for detecting patients in early stages with normal LV diameter and left systolic EF at the lower limit of normality as has been described before [21, 22]. They showed abnormal diastolic pattern according to TDI [23, 24].

Echocardiographic data have some interesting characteristic features, probably related to the genetic defect that we could not contrast in the literature because of lack of data. We have not found any information between septal knock as a preclinical indicator in bibliographic search due to lack of clinical assessments associated with this entity (DCM and *BAG3*). This slight proto-diastolic movement in the apical interventricular septum has been described in patients without dilatation or at early stage of the disease. The meaning of this septal knock remains unclear; however, no conduction abnormalities were observed. Because this knock can be seen in preclinical stages, it therefore enables diagnosis before there is impairment in the cardiac function. In addition, we observed LV enlargement greater than that observed for other genetic defects associated with familiar DMC. Finally, LA enlargement, despite the abnormal diastolic pattern and the enlargement of the LV, does not match the expected relationship. Concretely, we have observed moderately dilated LV with non-enlarged LA. On the clinical side, the age of presentation is much earlier, with lower EFs than other DCM-related genetic defects. In addition, in contrast to what it is described with other genetic DCM such as those attributed to *LMNA* variations, we have observed that these patients respond better to treatment with ACEI and beta-blockers, improving systolic function.

The BAG3 protein is incorporated into mature cardiac myocytes within the sarcomeric Z-disk and is thought to fulfill co-chaperone functions by regulating the family of heat shock protein 70 (Hsp70). Hsp70 proteins are crucial elements of the cell's machinery for protein folding and for protection of cells from stress [10, 25]. In the Polish family described by Franaszczyk et al. [12], the authors suggested that compound *BAG3*-stress could trigger acute DCM symptoms. This hypothesis was based on a loosened apoptosis control mechanism. The specific mechanism of altered Z-disc assembly caused by the DCM-associated *BAG3* pathogenic variations is not clearly understood, but a knockdown of *BAG3* in cardiomyocytes induces rapid myofibrillar degeneration and Z-disc disruption under the condition of mechanical stress [12]. This suggests that *BAG3* might play a pivotal role in Z-disc assembly during myofibrillogenesis. In fact, knockout mouse models show degeneration of muscle fibers with apoptotic nuclei in the striated muscles, resulting in skeletal myopathy and cardiomyopathy [26]. This fact could explain that pathogenic variation in the *BAG3* gene leads to symptomatic DCM in the early forties, as occurred in our family. In addition, only 2 *BAG3* truncated mutations associated with DCM have been reported (p.Trp26Ter -rs535344112- and p.Arg123Ter -rs387906875-). The frequency of both mutations was 0.0008991% (1/111226) and 0.001652% (2/121084), respectively. This suggests the rarity of this kind of mutation in the population and, in consequence, their potentially highly pathogenic role. Our results are similar to those reported by Norton et al. [7], who described a wide range at the onset of the disease (from 21 to 64 years old). They also reported high disease variability, from asymptomatic patients to heart transplantation, as happened with our cases [7]. The triggers for enhancing the gene expression are unknown, but viral infection was shown to induce the expression of *BAG3*. Homma et al. described that BAG3-deficient mice exhibit fulminant non-inflammatory myofibrillar degeneration with apoptotic features, as has been shown in our proband [26]. In the study family, four relatives presented the disease after a pneumonia episode. However, in all these cases the symptoms as well as evolution of the disease were similar to other affected relatives without any infection. Despite this fact, further molecular studies should be performed to clarify this hypothetic point.

The penetrance of the disease is closely related to patient age as already seen in other cases of DCM. Interestingly, Franaszczyk et al. [12] propose that BAG3 truncations result in an earlier onset of symptoms, although the severity of the disease is stronger for missense mutations, in contrast to what is described in *LMNA* [27]. In the study family, the median age of DCM patients was 43 years, but those undergoing treatment with drugs are showing clinical improvement. Close follow-up is necessary to discern the pathogenic role of this genetic variant and familial disease evolution, but previous results lead us to think that the observed truncated mutation has a degree of pathogenicity that coincides with that proposed by Franaszczyk et al. [12]. Concerning this point, NGS analysis in other relatives did not show any other genetic alteration.

It is possible that other disease modifiers, either genetic or environmental, might have influenced the phenotypic expression in this family [28]. Globally, these observations suggest that pathogenic genetic variants in *BAG3*, despite intrafamilial variability, demonstrate some phenotype specificity. Studying this subtle genotype-phenotype correlation may facilitate a better understanding of the mechanism of the disease in DCM and other BAG3-opathies associated with peripheral muscle weakness or neurologic findings [29, 30].

Current guidelines recommend genetic testing only of main genes associated with the disease, but *TTN* is not included due to recent association with DCM. Our study is in concordance with other publications, supporting the importance of performing comprehensive genetic studies in DCM families [31]. The lack of comprehensive genotype-phenotype correlation studies in DCM families raises controversies among cardiologists about whether performing SCD risk stratification in patients diagnosed with DCM aids in early identification and prevention of SCD.

### Study Limitations

As described, the main limitation of this study is a lack of large follow-up of patients. However, during the study it was observed that some patients have experienced a systolic function improvement with treatment. The echocardiographic data require further studies to confirm our results.

## Conclusions

We have assessed a large family suffering from DCM whose members carry a novel pathogenic variation in the *BAG3* gene. Frameshift variations in this gene correlated with a severe form of the disease, mainly in younger individuals. Additional comprehensive genotype-phenotype correlation studies should be performed in other large families suffering of DCM due to *BAG3* mutation as well as molecular studies to clarify cellular pathophysiology of DCM associated with BAG3. In this way, we propose echocardiographic characteristics not associated so far with *BAG3* which they should be evaluated in future clinical trials. The information will improve genetic counseling to families and help clinicians to adopt a proper treatment in each case to prevent acute HF episodes that can lead to death.
